# General Overview on Nontuberculous Mycobacteria, Biofilms, and Human Infection

**DOI:** 10.1155/2015/809014

**Published:** 2015-11-04

**Authors:** Sonia Faria, Ines Joao, Luisa Jordao

**Affiliations:** National Institute of Health Dr. Ricardo Jorge, Avenida Padre Cruz, 1649-016 Lisboa, Portugal

## Abstract

Nontuberculous mycobacteria (NTM) are emergent pathogens whose importance in human health has been growing. After being regarded mainly as etiological agents of opportunist infections in HIV patients, they have also been recognized as etiological agents of several infections on immune-competent individuals and healthcare-associated infections. The environmental nature of NTM and their ability to assemble biofilms on different surfaces play a key role in their pathogenesis. Here, we review the clinical manifestations attributed to NTM giving particular importance to the role played by biofilm assembly.

## 1. Introduction

The genus* Mycobacterium* includes remarkable human pathogens such as* Mycobacterium tuberculosis* and* Mycobacterium leprae*, both members of the* M. tuberculosis* complex (MTC), and a large group of nontuberculous mycobacteria (NTM). The NTM group comprises more than 172 different species with distinct virulence features (http://www.bacterio.net/mycobacterium.html). NTM usually exhibit saprophytic, commensally, and symbiotic behaviors [1]. Although mostly nonpathogenic, NTM are important environmental opportunistic pathogens of humans and animals, including poultry and fish [2, 3]. The NTM are ubiquitous in nature sharing with humans and other animals a wide variety of habitats. Over the past decades, NTM have been isolated from natural resources such as water, soils, domestic and wild animals, milk and food products and from artificial or built resources, such as home water distribution systems like showerhead sprays and sewers [2, 3].

The notification of NTM infection cases is not mandatory, in opposition to tuberculosis (*M. tuberculosis* infection). This fact hampers the accurate knowledge of the impact of NTM infections on public health. Nevertheless, it is largely accepted that in western developed countries the prevalence of these infections is growing as tuberculosis follows the opposite trend [4]. The impact of NTM infections has been particularly severe in immune-compromised individuals being associated with opportunistic life-threatening infections in AIDS and transplanted patients [5, 6]. Nevertheless, an increased incidence of pulmonary diseases [7, 8] and healthcare-associated infections (HAI) in immune-competent population highlighted the importance of NTM on human health [9, 10]. Medical devices related infections, one group of HAI, usually linked with bacterial biofilm proliferation on these materials, have been described for NTM [11]. The ubiquitous nature of NTM even allows their persistence within biofilms on other healthcare unit surfaces, such as water pipes. Biofilm persistence within healthcare units represents a threat to human health since it favors the onset and spread of HAI [12].

Biofilms are described as colonies of microorganisms attached to each other and to a surface, in an irreversible mode [13]. During biofilm development, bacteria suffer several changes in their phenotypic state forming a heterogeneous, dynamic, and differentiated community. They are part of a successful bacterial survival strategy in severe environments, since biofilm provides protection against environmental stressors, for example, antimicrobial agents and disinfectants [14–16]. For this reason, NTMs biofilms are an important research topic in mycobacteria pathogenesis [17].

## 2. Epidemiology and Clinical Manifestations of NTM Infections

Although being worldwide distributed, NTM experience significant geographic differences in terms of species incidence largely explained by the environmental nature of these microorganisms. Bacteria from the* Mycobacterium avium* complex (MAC) predominated in most western and European Union (EU) countries, followed by* M. gordonae* and* M. xenopi* [4]. In EU countries, another member of MAC (*M. intracellulare*) and the rapid grower* M. fortuitum* are the next most frequent NTM isolated [18]. In the United States of America, MAC members are most often isolated, followed by* M. kansasii* and* M. abscessus* [5].

A study conducted in Saudi Arabia (Middle East) rendered an opposite picture to that described above. The major species isolated were* M. abscessus*,* M. fortuitum*, and* M. intracellulare* followed by* M. kansasii*,* M. gordonae*, and* M. avium* [19]. The same is also observed in India where* M. fortuitum* is the most frequently isolated NTM [20]. In other eastern Asiatic countries located between Singapore (west) and Japan (east), bacteria from MAC account for majority of infections [20]. This peculiar aspect of NTM represents a challenge in terms of infectious disease management. In different geographic spots, the etiological agent responsible for the infection will be different requiring completely different therapeutic approaches.

The accurate diagnosis requires the identification of the etiological agent at the species level. The lack of a universal identification algorithm together with the ability of NTM to affect different organs exhibiting an age dependent tropism makes this a difficult achievement. In adults, chronic lung disease, bone infections, joints, and tendons are the most frequent pathologies. In children, skin and lymphatic nodes are the most affected organs. The majority of NTMs are nonpathogenic to humans being frequently opportunistic infectious agents. Rapid grower mycobacteria (RGM) and slow grower mycobacteria (SGM) exhibit a differential epidemiology of infection. Usually, RGM infections are mostly cutaneous and osteoarticular, whereas SGM infections are located on lungs and lymph nodes [6].

Among the pathologies caused by NTM disseminated infections were the first to attain the medical community attention. Initially, disseminated NTM infections were reported, almost exclusively, in severely immune-compromised individuals, where disease progression can be very rapid and even fatal. MAC members were the first to be identified as etiologic agents of opportunistic infections among AIDS patients back in the 1980s [21]. Until today, MAC accounts for the overwhelming majority of cases with* M. avium* being responsible for 90% of the cases [8, 21–24]. Excluding MAC,* M. kansasii* is the most common etiologic agent of these infections. However, other NTM, such as* M. scrofulaceum, M. gordonae, M. haemophilum, M. genavense, M. celatum, M. conspicuum, M. xenopi, M. fortuitum, M. marinum, M. malmoense,* and* M. simiae*, have also been described as causing the pulmonary or disseminated disease in AIDS patients [23, 25–34]. There are also reports of mixed infections or infections caused by more than one NTM [35].

Disseminated NTM infections have also been described in other immune-compromised populations, such as patients with cystic fibrosis, chronic obstructive pulmonary disease, renal failure, transplant recipients with chronic corticosteroid use and TNF-*α*, and leukemia [3, 18]. The frequency of these infections remains on the rise, in immune-compromised patients, due to the administration of immunosuppressive drugs or genetic causes [6].

Many RGM are often involved in postsurgical or posttraumatic infections, the most common being* M. fortuitum*,* M. chelonae*, and* M. abscessus* [5, 6]. HAI of skin and soft tissues due to these three species are caused by prolonged use of intravenous or peritoneal catheters, liposuction, postmammoplasty surgical wounds, cardiac bypass, and postlaser surgery cornea infections [36–41]. However, cases involving new species, such as* M. goodii* and* M. massiliense*, have recently been reported [42–45].

The respiratory infections, namely, affecting the lungs, are the most frequent. Patients with structural lung diseases such as chronic obstructive pulmonary disease, bronchiectasis, cystic fibrosis (CF), pneumoconiosis, prior tuberculosis, pulmonary alveolar proteinosis, and esophageal motility disorders are more prone to NTM infections [5]. Children with CF are usually affected by* M. abscessus* and closely related species, and adults are more frequently affected by members of the MAC [6]. Although SGM are largely responsible for lung and lymph node diseases, RGM, particularly* M. immunogenum*, are most frequently involved in hypersensitivity pneumonia [46, 47].* M. bolletii* (now reclassified as* M. abscessus* subsp.* bolletii*) is an emerging pathogen responsible for respiratory tract infections in patients exhibiting compromised respiratory function, being very resistant to clarithromycin [48, 49].

Localized cervical lymphadenopathy is more common in children aged between one and five years old [50–52] being rarely observed in adults in the absence of HIV infection [5]. In recent decades, a major shift in the etiology of cervical lymphadenitis was observed.* M. scrofulaceum*, previously regarded as the predominant cause of the disease, has become quite rare, with 80% of cases being attributed to MAC [52]. In Scandinavia, United Kingdom, Northern Europe, and Israel, the incidence of this disease is increasing due to* M. malmoense* and* M. haemophilum* [53–56]. The number of new NTM species isolated from lymph node biopsies has been reported to be increased, namely,* M. lentiflavum* and* M. bohemicum* [57, 58].

While virtually all NTM species have been described as etiologic agents of skin diseases, the species most frequently causing localized infections of the skin and subcutaneous tissue are* M. fortuitum*,* M. abscessus*,* M. chelonae*,* M. marinum,* and* M. ulcerans* [5, 6]. A common feature in these infections is exposure to contaminated water or infected fish. Although most skin lesions caused by infected fish are due to* M. marinum*, cases of infection by* M. fortuitum* and* M. chelonae* have been described [6].* M. ulcerans* is the causative agent of Buruli ulcer, the most common mycobacterial disease following tuberculosis and leprosy [59, 60]. Cases of mixed infections by NTM have been reported, as well as outbreaks which are associated with invasive procedures such as intramuscular injections and mesotherapy [61–68]. Outbreaks in postoperative surgical settings, such as cosmetic therapies, have also been described [43–45, 69].

## 3. Clinical Manifestations Associated with NTMs Biofilms: A Particular Case

The association between NTM biofilms and human disease is still recent, being unequivocally proven only for few species [3]. As for many other aspects, the link between biofilm and infection was first established for* M. avium*. This bacterium is able to proliferate within showerheads as biofilm from which infectious droplets could be released during a hot shower [70]. A similar process has been described for the waterborne pathogen* Legionella pneumophila* showing that this is not a mycobacteria exclusive persistence/infectious strategy. The isolation of NTM from biofilms collected in other water systems, namely, present in healthcare units, supports this route of dissemination of HAI by NTM [70, 71].

Another group of HAI with growing relevance is the medical device associated infections. The microscopic examination of a prosthetic aortic valve removed from a patient allowed the identification of a structure composed of NTM, a thin fibrin matrix layer associated with CD38 macrophages and a low number of platelets consistent with a biofilm. In this case, the prosthetic valve endocarditis had as etiologic agent the RGM* M. fortuitum* [72]. Another bacterium of this group* M. abscessus* subsp.* massiliense* was linked to a postchirurgical aesthetic breast implant case. The patient had a simultaneous infection of the right gluteal region being an example of surgical site contamination by NTM, and possible contamination of chirurgical material [73]. The ability of NTMs to form biofilms also contributes to the pathogenesis of catheter-related bloodstream infections [12]. A study of CF patients with lung infection demonstrated that* M. abscessus* grows in microcolonies similar to a biofilm. The observed phenotype is attributable to the cord growth formation of this NTM [74].

Although few in number, the etiology of this clinical manifestations is of great concern for public health. The ability of NTM to persist within biofilm on medical devices, both inside and outside the human body, together with the increase use of invasive diagnostic and treatment procedures, envisages an increase of these reports in the newer future.

## 4. NTM Transmission and Environment

In general, the opportunistic mycobacteria may become pathogenic only in certain specific conditions. As so, since they are environmental species, it is common to find them colonizing the respiratory, gastrointestinal tract and skin, not being a source of infection [71]. The presence of opportunistic pathogenic species (e.g.,* M. avium*) in a clinical sample is not sufficient to attribute the classification of causal agent of the disease. In these cases, it is mandatory to identify the same NTM species both in the infection source and the patient [75].

The transmission of NTMs can be established through environmental source or clinical settings to the patient, rather than between patients [75, 76]. Humans could be infected by NTM present in aerosol droplets by inhalation, ingestion, or trauma events [3, 77]. The environmental sources of NTM most relevant are water, soil, and dust. A characteristic of mycobacteria, high hydrophobicity, is of key importance for the success of infection. Hydrophobicity favors aerosolization and consequently mycobacteria transmission and onset of infection, for example, in alveoli. On the other hand, hydrophobicity favors bacterial adhesion to surfaces promoting biofilm assembly which could work as a disinfectant and antibiotic resistance mechanism [3]. This aspect will be detailed in another section of the paper.

In general, contamination of medical equipment by* M. tuberculosis* has its origin in patients. However, in the case of NTM, the source of contamination resides mainly in tap water and can occur, among other possibilities, through solutions used in the disinfection of endoscopes and during automatic washing. In the last case, contamination can result from the presence of a biofilm inside the instrument [78]. Since these devices are difficult to sterilize, they may contaminate the sample during collection leading to pseudoinfections [79].

## 5. Characteristics of Biofilm-Grown Bacteria

Biofilm assembly is a dynamic and complex process divided in several phases, including reversible attachment, irreversible attachment, maturation, and dispersion [16]. The attachment phase is dependent on electrostatic interactions between bacteria and the surface. Bacteria only attach to a surface if they sense stable nutrient concentrations, beneficial temperature, and oxygen level [16]. During biofilm assembly, bacteria secrete a matrix containing polymeric substances such as polysaccharides, lipids, and nucleic acids. The extracellular matrix plays a key role in biofilm architecture allowing the assembly of a complex three-dimensional structure [16, 80]. Bacteria within a mature biofilm are completely differentiated, achieving their maximum replication rate [81]. When the nutrient levels decrease, or bacteria density increases in a certain area, bacteria can rapidly disperse and colonize new spaces, in search for better conditions [16]. Quorum-sensing (QS) also known as bacteria cell-to-cell communication mediated by autoinducer molecules plays a regulatory role in this process being of particular importance on both attachment and dispersion phases [82, 83].

In the case of NTM pathogenesis biofilm assembled within healthcare units plays an important role, being more common on water distribution systems and plumbing pipes [84–87]. Evidence mounts in support of the observation that tap water functions as a privilege channel for human colonization and/or infection by NTM [66, 79, 88–90].* M. avium* is one of the most studied NTM regarding biofilm production. This* Mycobacterium* is able to assemble biofilms even when incubated only with water explaining its presence on showerheads, water distribution systems, and clinical settings [91, 92]. Biofilm assembly was even exacerbated in the presence of divalent cations and carbon sources [91].

As mentioned before, bacteria within biofilms exhibit an enhanced resistance to antimicrobial agents, which could be 10- to 1000-folds higher when compared to planktonic bacteria [93]. This phenomenon accounts, at least in part, for an increase in bacterial virulence, being of particular concern for bacteria naturally resistant to antimicrobials such as NTM. Resistance to disinfectants, such as chlorine, has been reported as being one of the factors responsible for the colonization, persistence, and replication of NTM within drinking water distribution systems [3, 75, 94]. Biofilm organization hampers NTM eradication with common decontamination practices and is relatively resistant to standard disinfectants [91], such as chlorine, organomercurials, and alkaline glutaraldehydes [73, 95]. The mechanisms responsible for this phenomenon are less understood, but it is known that biofilm growth depends on bacteria-surface affinity and environmental conditions.* Mycobacterium fortuitum* has a higher biofilm development affinity in stainless steel, polyvinyl chloride, and polycarbonate rather than copper and glass [91].

In the case of antibiotic resistance, several mechanisms have been implicated in this virulence increase for bacteria in general. One of them is horizontal gene exchanges favored by the maximum proximity experienced by bacteria within biofilms [96]. This gene transmission is a major cause for bacteria survival [97] and can account for a high frequency of mutations responsible for antimicrobial resistance [98] mediated by triggering enzymatic production, modification of antibiotic target, or expression of efflux pumps [99–101]. Another mechanism is the appearance of persisters defined as phenotypically different bacteria exhibiting slower growth rate [102]. The persisters tend to be located in the biofilm areas with lower nutrients and oxygen concentrations [87]. For this reason, the phenotypic switch could be regarded either as bacterial survival strategy in a harsh environment or a virulence mechanism. The last option is explained by the decreased activity of the majority of the available antibiotics against nonreplicative bacteria [103].

For NTM, the boosting of antibiotic resistance promoted by biofilm assembly seems to be adaptive rather than genetic. When organized within biofilms,* M. avium* is transiently more resistant to antibiotics and antimicrobial agents. Nevertheless, bacteria recovered from biofilms lost resistance in a short period of time (e.g., 1 day) showing that despite being a SGM* M. avium* has a rapid metabolic adaptation rate [3]. This observation also suggests that biofilm induced antibiotic resistance might be attributed to a structural factor.

Being the scaffold of biofilm, extracellular polymeric matrix (EPS) is most probably involved in the emergence of antibiotic resistance. The self-produced EPS is also considered important for enhancing bacteria virulence. EPS builds a barrier that can inactivate antibiotic, delaying or preventing antibiotic penetration within the biofilm and recognition of their targets [104]. Although EPS composition is not well known even for the most studied NTM,* M. avium* [105], interspecies differences in EPS nature had already been reported [13].* M. smegmatis* EPS is constituted by free mycolic acid, glycopeptidolipids, and mycolyl-diacylglycerol.* M. abscessus* includes mycolyl-diacylglycerol,* M. marinum* lipooligosaccharides, and lipopeptides in* M. avium* subsp.* paratuberculosis* [105]. During the biofilm formation and its establishment, some genes related to the GPL biosynthesis were upregulated in* M. avium and M. smegmatis*, showing that the GPL synthesis and biofilm formation are intimately connected [106]. For* M. smegmatis*, it has also been shown that mycolic acids synthesis is increased in the presence of antibiotics suggesting a role in the emergence of drug resistant persisters [107]. In addition to lipids, the presence of other factors such as GroEL1 [108], protein kinase [109], and iron [110], or the lack of others, for example, polyphosphate deficiency, affects biofilm formation, matrix composition, and structure [111].

The existence of GPL in the cell wall outermost membrane of* M. smegmatis* and* M. avium* is associated with the ability to form biofilms and affects other proprieties such as colonies morphology, sliding motility, and immune modulation [112]. On the opposite,* M. tuberculosis* outermost layer, called capsule, is composed of phenolic glycolipids (PGL), phthiocerol dimycocerosates, and lipooligosaccharides [106, 113].

The cell-surface structures, such as pili, may have an important role in biofilm formation and surface attachment, like on some other bacteria [114]. Considering NTM, there are no studies available that correlate the existence of pili and surface adherence. Moreover, most of the published studies on biofilms were conducted on the model organism* M. smegmatis* and although NTM are devoid of flagella [115], it has been shown that the genetic requirements for sliding motility on agar surfaces and biofilm formation are similar [116]. A relation between sliding and biofilm assembly has also been established for* M. chelonae* and* M. fortuitum* [117].

## 6. Methodologies Used to Study Biofilms

Most of the studies in the biofilm field are focused on the identification of factors involved in the first phase of biofilm assembly: attachment. However, the last and less understood phase of biofilm assembly has also been the focus of several studies [118]. The methodologies followed are diverse and goal oriented.

The most common method used for following biofilm assembly* in vitro* is the microtiter plate test, which allows the observation of bacterial adherence on abiotic surfaces [119]. Stain techniques with crystal violet allow the visualization of biofilm and its quantification through spectrophotometry measurement. The microtiter plate test is the cheapest and less labour-intensive method [119, 120]. The ring test, Congo red agar, and resazurin assay are other techniques based on staining procedures coupled with spectrophotometric methods used for biofilm study [121–124].

Another technique that has been used is the microfermentor test that generates abundant biomass. This method have the advantage of allowing the extraction of nucleic acids and proteins, providing more information on biofilm assembly [118, 125]. For example, adhesins necessary for irreversible surface adhesion have been identified by genetic studies. However, the experimental support for the role played by these proteins in cell-surface interactions is still missing [126]. The use of cutting-edge technologies like next generation sequencing (NGS) and RNA sequencing to biofilms of different microorganisms is opening new perspectives [127–129]. The study of gene expression has become a major interest during the last decade, because it reveals important data on how bacteria sense and respond to various environments [127].

Atomic force microscopy (AFM) has also been used in the field [130]. This method presents a sensitive tool to study bacterial adhesion to surface [126]. Additionally, AFM allows the study of bacteria morphology [131] and surfaces with high resolution. This technique requires minimal sample preparation and allows the acquisition of 3D images of the surface ultrastructure in physiological conditions [126]. The huge potential of this technique could be enhanced by combination with confocal microscopy [132]. Since AFM imaging has raised several problems [132], other techniques have been used for this purpose, as scanning electron microscopy (SEM), transmission electron microscopy (TEM), fluorescence microscopy, or confocal laser scanning microscopy (CLSM) [133–136].

Fluorescence microscopy is a noninvasive method to assess biofilms, for example, the reactivity of an antibiotic in a biofilm [137]. Confocal laser scanning microscope is an optical microscopy technique, useful for the study of more thick samples [120]. This technique has been also important to analyse antimicrobials action; however, it has restricted magnification [137, 138]. Other possible techniques are cryo-SEM and environmental SEM (ESEM), where samples do not need to be dehydrated [138]. On cryo-SEM the sample is frozen with liquid nitrogen during the imaging; however, micrographs have less resolution compared to SEM or TEM [138].

The development and standardization of methods to evaluate minimal inhibitory concentrations (MIC) of antibiotics against biofilms is a hot topic in biofilm research and clinical practice. Increased antibiotic resistance by bacteria within biofilms required the design of different therapeutic schemes and the determination of MICs is the first step towards success. An assay to evaluate biofilm susceptibility to biocides known as MBEC (MBEC Biofilm Technologies Ltd., Calgary, AB, Canada) system has been developed. A unique 96-well plate with pegs projecting down from a plastic lid has been designed to evaluate antibiofilm activity of a battery of drugs in parallel [139]. Each well can be used to test a different antibiotic concentration, mimicking the MIC method used for evaluating antibiotic susceptibility of planktonic bacteria. The comparison of biofilm and planktonic bacteria susceptibility to antibiotics is one of the major advantages of this methodology concerning clinical applications [17].

## 7. Final Remarks

**Figure 1:**
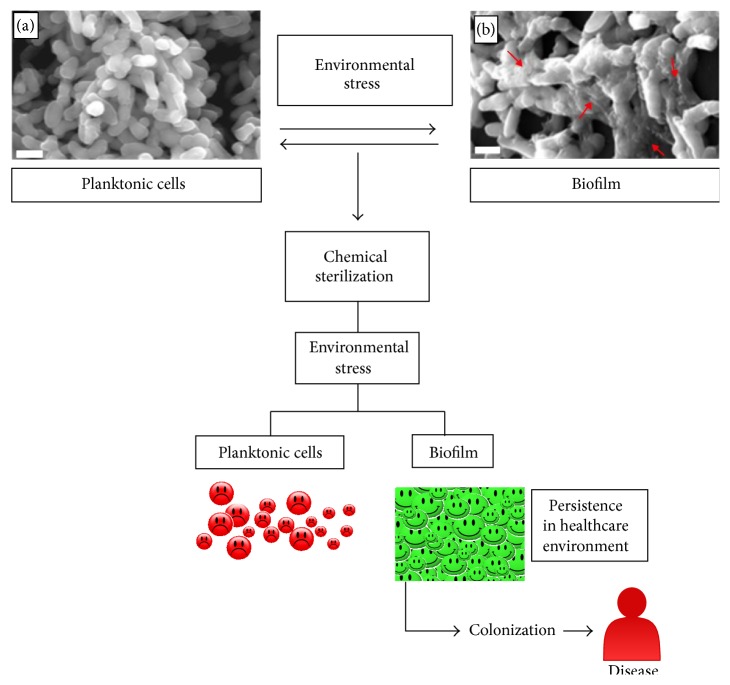
NTM persist in a steady state between planktonic (a) and biofilm (b) within healthcare units and on medical devices. Biofilm assembly is triggered by environmental stress. Bacteria organized in biofilm exhibit a different structure being notorious, increasing the amount of extracellular matrix (red arrows). Another feature of these bacteria is the increased resistance to chemical sterilization, which leads to persistence within healthcare units, host colonization, and onset of disease. (Red circles: dead bacteria; green circles: live bacteria; scale bar 1 *μ*m.)

NTM are emergent pathogens with growing impact on human health. Their condition of environmental bacterial enables them to persist in a wide range of conditions. In addition, NTM once submitted to environmental stress can assemble biofilms (Figure 1), enhancing their resistance to antimicrobial agents, persistence within healthcare units, and the probability to colonize and cause disease in humans. This scenario is particularly problematic since 80% of infections are caused by bacteria organized in biofilms which are refractory to many therapeutic agents currently in use.
